# Pursuing impact in research: towards an ethical approach

**DOI:** 10.1186/s12910-022-00754-3

**Published:** 2022-04-06

**Authors:** Kristine Bærøe, Angeliki Kerasidou, Michael Dunn, Inger Lise Teig

**Affiliations:** 1 Department of Global Public Health and Primary Care, University of Bergen, Postbox 7804, 5020 Bergen, Norway; 2 Ethox Centre, Big Data Institute, Li Ka Shing Centre for Health Information and Discovery, University of Oxford, Old Road Campus, Oxford, OX3 7LF UK; 3 Centre for Biomedical Ethics, National University of Singapore, Singapore, Singapore

**Keywords:** Impact of bioethical research, Responsible research and innovation (RRI), Translational ethics, Activism, Self-reflexivity, Ethical framework

## Abstract

**Background:**

Research proactively and deliberately aims to bring about specific changes to how societies function and individual lives fare. However, in the ever-expanding field of ethical regulations and guidance for researchers, one ethical consideration seems to have passed under the radar: How should researchers act when pursuing actual, societal changes based on their academic work?

**Main text:**

When researchers engage in the process of bringing about societal impact to tackle local or global challenges important concerns arise: cultural, social and political values and institutions can be put at risk, transformed or even hampered if researchers lack awareness of how their ‘acting to impact’ influences the social world. With today’s strong focus on research impacts, addressing such ethical challenges has become urgent within in all fields of research involved in finding solutions to the challenges societies are facing. Due to the overall goal of doing something good that is often inherent in ethical approaches, boundaries to researchers’ impact of something good is neither obvious, nor easy to detect. We suggest that it is time for the field of bioethics to explore normative boundaries for researchers’ pursuit of impact and to consider, in detail, the ethical obligations that ought to shape this process, and we provide a four-step framework of fair conditions for such an approach. Our suggested approach within this field can be useful for other fields of research as well.

**Conclusion:**

With this paper, we draw attention to how the transition from pursuing impact within the Academy to trying to initiate and achieve impact beyond the Academy ought to be configured, and the ethical challenges inherent in this transition. We suggest a stepwise strategy to identify, discuss and constitute consensus-based boundaries to this academic activity. This strategy calls for efforts from a multi-disciplinary team of researchers, advisors from the humanities and social sciences, as well as discussants from funding institutions, ethical committees, politics and the society in general. Such efforts should be able to offer new and useful assistance to researchers, as well as research funding agencies, in choosing ethically acceptable, impact-pursuing projects.

## Background

Broadly speaking, a research impact is a marked effect or influence associated with research. Research impacts can take many forms. First, we can distinguish between research impact within and beyond Academy. The first kind of research impact refers to publications, citations, presentations at Academic conferences, but can also encompass supervision and training of new researchers who will continue the same research tradition.

Our focus in this paper is on research impact that influences the world outside Academia. Most commonly, the non-academic impacts are associated with an ‘effect on, change or benefit to the economy, society, culture, public policy or services, health, the environment or quality of life’ [1].Although not all research is equally relevant for pursuing impact of this kind, a distinction cannot easily be drawn based on disciplines. Work in physics, chemistry, anthropology and moral philosophy, for example, can aim simply to understand or explain phenomena. The impact of such research may remain within an academic setting. However, research can aim at tackling an identified challenge. In that case, the setting for the work is normative in the sense that the research is instrumentally subordinated to an external objective; knowledge, data or evidence of some kind is needed in order to change the world as it is. Research outcomes can then be tested; for example, a new drug either works effectively in curing a disease or it does not. Other types of normative research can escape assessment of this kind, either there are several ways to specify the objective to test for success (e.g., to make an institution more ‘just’), or because the research itself rests on certain assumptions about the kind of knowledge relevant to a good society. When conducting research that cannot be straightforwardly tested using well-established assessment measures, researchers have more discretion regarding when to pursue societal impacts based on that research. This includes *normative and interpretive research* that involves explicit or implicit perspectives on how the world *should* be organised or what *matters* and ought to be described. This kind of research cuts across a broad range of *applied disciplines*, including ethics, public health, economics, political science and sociology.

We also need to distinguish between societal impact that occurs without any effort from the side of the researcher to pursuit impact beyond the Academy, namely beyond presenting and publishing one’s work in academic venues and channels, and practical, societal impact deliberatively pursued by a researcher (or a research team). The latter version that involves researchers’ deliberative intentions to impact, is what is of interest here. However, specific, predetermined impacts that are set by policy priorities but involve research input and evidence alongside interventions instigated by politically legitimate governing authorities (e.g., a strategy for eradicating malaria infections in an area) are separate from the kind of impact that we will focus on. In brief, we are concerned with activities initiated by individual researchers, or research teams that aim at the specific and deliberative pursuit of real-world changes based on their own research and worldviews.

Moreover, shaping an intention of producing impact does not guarantee actual impact; the pathways to impact are contingent on multiple contextual factors, including the motivation and capacities of stakeholders. Therefore, we should also make a distinction between forming an intention of creating social impact, which is within the researchers’ control, and in what way this impact unfolds, which might be less within the control of the researcher. In this paper, we understand ‘pursuing impact’ according to how researchers interact with their surroundings to realise an intended impact of their research.

Are there boundaries to how far researchers should go in pursuing impact? Our working hypothesis is that there are such boundaries and that they ultimately depend on an ethically justifiable and unjustifiable distribution of power at the intersection of producing and applying knowledge. We will approach this question further by discussing in more detail (1) what *impact* involves, (2) how researchers can pursue *impact*, (3) what *external drivers* push researchers to pursuit impact, and (4) which gap of knowledge *a researcher ethics for acceptable and non-acceptable pursuits of research impact* (as opposed to *a research ethics*) can contribute to fill. Finally, our discussion is embedded in a proposed framework for what a self-reflexive, ethical approach to establish such boundaries for *an impact-pursuing ethics* would look like. We limit our analysis to the field of bioethics. As bioethicists centre on theoretical and practical, ethical issues related to health and environment, one might expect bioethicists also to be especially attentive to pursuing impact in an ethical acceptable manner. While this is an empirical question, academic bioethicists are nevertheless in an advantaged position in terms of their theoretical and practical training when it comes to identify ethically relevant conditions for pursuing impact of normative research. Thus, we will suggest preliminary steps for how to establish a context-sensitive and dynamic framework that can promote reflexivity amongst bioethicists regarding potential boundaries to intentionally pursued impact of bioethical work. Similar steps can then be applied to specify ethical boundaries to pursuing impact of normative work in other disciplines, too.

## Main text

### Two approaches for pursuing practical impact based on research

There are two distinctive ways in which researchers can pursue societal impact: (1) directly implementing change in the world according to their own research and the normative worldview it represents, and (2) indirectly implementing change by promoting certain kinds of evidence emerging from research, making strong arguments for required changes in communication channels read by decision-makers, or by developing frameworks for tackling practical issues that translate normative ideas into practice. Regarding (1), the implications of pursuing impact are obviously embedded in culturally, socially and politically shaped contexts. These dimensions of society are conditioned by ideas of what constitutes a good life, typically founded upon broad political and social consensus. Enforcing research-based interventions (e.g. introducing new technology or a new policy) runs the risk of clashing with *cultural, social* and *political* values endorsed by those for whom the impact is intended.

Consider a scenario in which researchers convince global health donors to implement their research-based programme to fight a specific disease without any input or consultation with the relevant community. As a result, local healthcare systems could be distorted according to the required infrastructure (social), the population’s perceptions of their health-related needs might be set aside (cultural) and the local country’s ability to develop their own sustainable healthcare system might be sidelined (political). Conversely, researchers pursuing impact can constructively help communities eliminate arguably oppressive historical practices, such as female genital mutilation. However, even though the outcome of researchers’ influence would be deemed acceptable by most, the same social, political and cultural objections could still apply. In these various ways, researcher-pursued impact is connected to a wide range of possible outcomes and implications. Researchers pursuing impact of their own normative research are faced with not only significant degrees of ethical ambiguity, but also novel ethical issue on the ground that they might not be able to address or resolve, at least not directly and immediately.

To realising impact by way of (2), that is, by promoting certain kinds of evidence, robust arguments or normative frameworks, the outcome of knowledge production must be applicable and appropriate for the context it aims to improve. Ideally, evidence must both accurately describe what it claims to describe (i.e., the data must be ‘true’), and adequately capture a normative understanding of what needs to be improved (i.e., the scope of the data must be justified according to a goal). As researchers exercise control over the production of scientific findings, they influence what is considered relevant knowledge simply by claiming the superiority of certain empirical methods or by describing or measuring aspects of the world in particular ways instead of others. In this sense, research, as an institutionalised form of inquiry, is intimately connected to, and legitimised within, processes of social change and reform in ways that are distinct from other activities that produce evidence, arguments or frameworks [2].

### How researchers can pursue impact–and some potential pitfalls in doing so

To discuss the boundaries constraining how and when researchers *should* pursue impact, we first need to understand how they *can* do so. Most conspicuously, perhaps, researchers can proactively engage in practical changes by claiming authority based on their expertise and the force of their claims; they can participate in negotiations about which research initiatives and scientific approaches to fund, and which policies or interventions to implement, uphold or dismiss. On an uncontroversial account, researchers play an essential role in influencing societal changes by developing scientific knowledge and informing policymakers and public debate. However, it is not at all clear how and to what extent researchers should carry out societal changes via advocacy [3], such as convincing stakeholders or orchestrating or facilitating local solutions to global challenges according to their own research interests and normative ideals. This quandary occurs when researchers are not only expressing their views in public debate or advising governing authorities on invitation, but also engaging in and influencing societal changes in their capacities as researchers as opposed to elected politicians, representatives of organisational bodies or citizens*.*

When researchers take on the role of experts (self-imposed or according to the judgments of others), their epistemic authority in applying methods or discussing difficult issues may be (mis)taken for the authority to make competent, *political* decisions outside the academic context as well. If researchers deploy their own authority uncritically, they can end up distorting social and political systems. For example, otherwise well-functioning democracies can be turned into political systems in which unelected researchers define the aims for and means of change, subsumed and legitimated under the banner of ‘evidence’ or ‘knowledge’. However, in controlling methods and knowledge production, researchers can influence the evaluation of implemented policies in line with their own interests and values, assumed political ideals, or personal career ambitions. To avoid such negative impacts, researchers must—as a necessary but not sufficient condition—demonstrate a reflexive awareness of the limitations of what they can seek to change*.* An important distinction to bear in mind here is that researchers can have an intention to pursuing impact of their own knowledge productions, but also facilitate co-production of knowledge among multiple stakeholders to help bring about impact. If the impact is the goal and co-production only a mean, there are reasons to be skeptical to the pursuit of impact. However, as we will see below, involving stakeholders in co-production of knowledge when aiming for reasonable and supported solutions to change, may not challenge the appropriateness of the impact. Indeed, involving stakeholder in co-production early on in the research process, even already at the stage of defining the research question, can be crucial for realizing any impact at all.

To summarise: there are clearly limits to when it is ethically appropriate for researchers to change actual practices by allowing their own normative research conclusions to bypass socially, culturally and politically legitimate norms and institutions. At the same time, exactly where to draw these boundaries, and the ethical principles that ought to shape how impact is pursued within these boundaries, remains unclear. Arguably, this calls for less generalisation and more contextualisation and specification of *when* in decision-making processes it is appropriate to push impact, as well as *where* the impact is pursued.

### External drivers to pursuing impact

Powerful social forces are pushing researchers to pursue impact. *First*, there are political drivers. With the Sustainable Development Goals (SDG) 2030 agenda, for example, the United Nations has agreed on the need to tackle a variety of global challenges, such as reduction of poverty (SDG1), health inequalities (SDG3) and mitigation of climate change (SDG13). This focus on finding practical solutions shapes the involvement of everyone engaged in responding locally to global challenges, including the researchers whose role extends from producing knowledge to realising change.

*Second*, governments need to demonstrate to the public the value of investing in science [4], and funders are interested in seeing tangible outcomes of their investment. Funding bodies in the EU, UK and Norway for example, apply ‘impact’ as an assessment criterion for applications, which encourages researchers to design projects that will result in demonstrable impact. On a weak interpretation, the focus on impact encourages more reflectiveness among researchers regarding the outcomes of their work [5], and it offers leeway for including ethical awareness concerning the acceptability of pursuing impact of this outcome. On a stronger interpretation, researchers are encouraged to realise the potential influence of their research.

*Third*, researchers might have specific reasons for pursuing impact based on their research. Researchers in the health area might be deeply engaged in, and committed to, bringing about broad normative goal of improving people’s health and lives in general. Within bioethics, the emergence of accounts of research activity that are explicitly focused on ‘impact’ or ‘activism’ are becoming increasingly well-established and endorse such goals as a central part of the research activity [6, 7]. Whilst seeking to achieve such goals is difficult to dispute, those outlining these approaches clarify the need for careful attention to be paid to how such goals are set, how they are specified in different real-world contexts, and how impact or activist agendas are established in ways that enable the appropriate and legitimate translation of bioethical scholarship into bringing about practical change [8, 9].

Equally, researchers might be motivated to bring about impact by the promotion of their own value commitments, or the advancement of their careers. Although acting according to such interests is rational, it will not always be ethically or politically appropriate; the interests of researchers may clash with—and should not necessarily trump—other legitimate and complex interests within a society. Common to these forces is a blindness to what qualifies as ethically acceptable research-based impacts. Again, a focused exploration of what such assessment would amount to is urgently needed.

### Knowledge gap: no state of the art

In research ethics, i.e. the ethics of planning, conducting and reporting on research, there is no ethical standard that explicitly addresses and formulates the state of the art of impact-pursuing researchers. This appears to be because research ethics, as an academic field, has implicitly adopted the view that research activities worthy of ethical scrutiny conclude at the point at which the research project itself is completed. Notwithstanding this observation, there are distinct approaches that point to relevant elements that might comprise such ethical standards. Codes of ethics or ethical standards that promote academic integrity, researcher professionalism and participatory research approaches all address substantive elements of what could facilitate the development of such an ethics framework which emphasises the ethics of *researchers* rather the ethics of *research*.

For societal changes to be sustainable, knowledge production itself must be sensitive to the perspectives and needs of those who will implement and live with the change, that is, distinct communities/society at large. Accordingly, producing applicable knowledge that is fit to bring about change in the world calls for research that comprehensively integrates ‘what is’ knowledge with practical knowledge of what should be done in order to achieve a welcome change, According to Choi and Pak:

“Interdisciplinarity analyses, synthesizes and harmonizes links between disciplines into a coordinated and coherent whole.” [10] (p. 351). Moreover, involving stakeholders in co-production of knowledge extends disciplinary boundaries as “[t]ransdisciplinarity integrates the natural, social and health sciences in a humanities context” [10] (p. 351). These approaches aim to ensure the relevance, fairness and legitimacy of results by guarding against undue external influences, which, so the argument goes, is better placed to have legitimate impact in the world than through knowledge produced within disciplinary silos*.* However, as pointed out in the literature, they also involve overcoming barriers to change in order to be successful [11–13]. Just as importantly, the mere fact that cross-, inter-, or trans-disciplinary modes of research have been undertaken does not provide, without further argument, a persuasive reason to think that the impact pursued through this research will be ethically defensible, nor that it will be straightforward to implement. Even though the resulting knowledge of such processes satisfies the social value requirement, trying to implement this knowledge can involve practical concerns that render any attempt on pushing the impact of the research ethically questionable. For example, imagine a philosopher who has carefully thought through a very detailed questionnaire to help doctors test that patients have thoroughly understood every sentence in a consenting form to make sure their consent is truly ‘informed’ when it comes to choosing an invasive treatment. Implementing such time-consuming testing of understanding to ensure actually ‘informed consent’ of patients rather than carry it out as merely a formal procedure, will reflect social value. On the other side, insisting on implementing such a resource and time-consuming procedures in emergency care units, for example, where delays can lead to poorer health or death of the patients, as well as other patients when human resources are scare, would be hard to justify. Cultivating attentive concern about such practical and political constraints on what brings out social value requires a researcher ethics rather than an ethics for research.

Similarly, the Responsible Research and Innovation (RRI) movement [14] aims to foster sustainable, socially acceptable research and innovation through participatory research, and it offers valuable insights into how to produce knowledge that will be perceived as legitimate and applicable. Engaging in participatory research, in which stakeholders and researchers develop the research agenda and conclusions collaboratively, is one means for researchers to become reflective about the justified impacts of their research [13, 15]. However, researchers’ contributions to participatory research programmes can skew the outcomes if they assume the role of experts, as noted above. Moreover, the RRI movement does not address a comprehensive, targeted ethical approach for researcher-pursued impact that could be applied to all the phases of research and application: identifying a problem, designing a research project, providing evidence and implementing a solution. Again, a researcher ethics calls for targeted attention to *what* research one should pursue, and *when*. It calls for fostering reflexivity amongst researchers to enable scrutiny of what distinguish justified from unjustified impacts. RRI does not target this kind of reflexive activity.

Such reflexivity is, however, central to a proposed ‘translational ethics’ approach [16]. This elaborated version of translational ethics offers a comprehensive strategy for *framing* discussions regarding the conditions for acceptable pursuits of impact [16]. It requires that whenever researchers are bridging the gap between theoretical, ethical work and practice, each step of identifying concerns, establishing normative knowledge, implementing normative knowledge and evaluating actions or policies should be carefully, and coherently, justified and organised so that the ‘ethics of doing ethics’ is openly discussed, and stakeholders are given reasons to confer legitimacy on the whole process [17]. As an analytical framework, translational ethics provides a broad and useful frame within which to structure the required reflexivity to support an ethics for researchers who are pursuing impact. Importantly, this analytical framework is context-sensitive and puts the burden of providing justification on the researchers, but it does not provide any resources with which to identify substantial limitations to appropriate impact [18]. According to our working hypothesis, such assessments require support from substantive ethical and political discourses that address the issue of distribution of power at the intersection of producing and applying knowledge. In addition, it also calls for empirical investigation and concrete systematised knowledge about how impact in the field of bioethics actually can be obtained.

We have now proposed some possible ways forward to think about orientating ethical thinking about research impact. However, broadly speaking, the research community remains focused mainly on the ethical conduct of research, with limited attention paid towards the ethical dimensions of translating research (even research conducted across disciplines and in ways that adopt participatory methods) into real-world contexts. With global challenges and national research institutions calling for ever-increasing impact, it becomes urgent to direct attention to how the impact agenda in research ought to be fostered in ethically justifiable ways, and how researchers can scrutinise acceptable and unacceptable ways of pursuing impact within their own activities. An overarching, context-sensitive framework of ethical concerns regarding the transitional role of academics (i.e., from knowledge producers to impact producers) remains elusive. Being aware of our own limitation as an authoritative power to claim what this ethics should look like, we would like to propose certain structural conditions, we believe ought to be in place for such a cutting-edge decision-making process to function in the field of bioethics.

### Initial steps towards an ethical approach to pursuing impact

An approach to establish an ethics for pursuing research impact should be based on a legitimate process for shaping practice at the intersection of ethics, politics and expertise knowledge. This means that stakeholders (i.e. researchers, research funders, members of research ethics committees, politicians and any member of the affected society) have reasons to invest in the articulation of fair conditions for the development of this ethics framework. What would fair conditions for developing this ethics look like? We propose a four-step process that can establish the basic ethical foundations of how the pursuit of impact in research can and should be orientated. These steps are designed in such a way so as to allow for all stakeholders to consider, deliberate and potentially revise. This design reflects a general ethical concern for the moral equality of stakeholders, which we believe is important for this (and other) ethics framework.

Initial steps of an ethical approach for pursuing impact in research:

1. Explore the concept of impact and develop a theoretically and empirically informed conceptual framework of conditions for pursuing research-based impact in general and the field of health in particular. This step helps identify the kinds of action potentially involved when researchers are pursuing impact.
2. Explore empirically the structural opportunities available to researchers in translating their research into practice and the challenges associated with obtaining such support. This step helps identifying how researchers themselves navigate to have impact in relation to structural pathways and detect challenges as perceived from researchers own perspective in doing so.
3. Investigate, based on a proposed normative, theoretical framework, which ways of pursuing impact are acceptable and which are not, and to present the results as an ethical framework that reflects the scope and content of ethical challenges potentially encountered when pursuing impact. This step helps integrate the theoretically and empirically informed findings in 1)-2) with a developed framework structured by of existing literature on ethical and politically acceptable/unacceptable actions.
4. Organise scientific and popular dissemination of 1–3) to enable debate, critical scrutiny and potentially conferred legitimacy among stakeholders.

#### Step 1

What are we actually talking about when we talk about impact of research? As a first step, it is necessary to establish what the kind of ‘impact’ researchers can pursue amounts to in practice. And, it is important to determine how such impact should be conceptualised. We suggest there is a need to *explore the concept of impact and develop a theoretically and empirically informed conceptual framework of conditions for pursuing research-based impact in general and the field of health in particular.* This will require an interdisciplinary approach that combines philosophical analysis based on descriptive and normative theories with empirical research on real world experiences and perspectives. When conceptualising the 'impact' of normative health research, for example, we can take a generic model of impact as our point of departure (for example [4]), and explore the relevancy of a sociologically approach to 'impact' (for example [19]) when modifying it for the healthcare field and accounts of academic activism when relating it to bioethics [8]. While discussing the impact involved in producing knowledge or evidence within the sciences, it would be relevant to draw on philosophy of science literature addressing the contextualisation of the production of scientific knowledge [20] and different modes of knowledge production [21].

#### Step 2

What opportunities do researchers have for translating their research into something that can have a societal impact? As a second step, it is necessary *to investigate the structural opportunities researchers have for translating their research into practice and the challenges associated with obtaining such opportunities.* Determining the causal relationships involved in obtaining impact is open to a variety of possibilities; effects can occur coincidently or can be intentionally and strategically pursued by researchers, funders, or others. However, intentions of creating impacts are no guarantees for success; contextual factors can complicate, distort or undermine the impact. In terms of structural factors, researchers operating at the intersection between scientific knowledge production and societal practice have opportunities to contribute with impact of their own research interests by influencing how political decision-making processes are organised, how implementation is carried out and, on what criteria are used to evaluate those. So far, the researcher community itself has not been very engaged in undertaking empirical explorations about when, where and how this impact is being played out in real world settings. Models of how 'impact' functions generally [4] and frameworks aiming to capture various kinds of research impact (e.g. [22]), do provide us with a helpful background for grasping the structural forces at work. To make this understanding relevant for distinct fields of research, however, these models and frameworks must be operationalized according to the specific conditions of both the research area and the context of research in question. Here, empirical research is called for. Empirical research on structural conditions and challenges can be reported in accordance with suggested standards for assessing empirical bioethics [23], which at least in part involves clarifying how normative theories are integrated with the empirical findings.

When conceptualising the transitional movement involved in moving from research activities themselves to pursuing actual impact, an approach can benefit from exploring structural understandings of discretion [24] and judgment [25], and an analysis of researchers' power to make their own decisions according to a parallel account of doctors' professional autonomy [26]. The descriptive findings in this step can in turn be fed into normative analysis in step 3).

#### Step 3

How should researchers act when pursuing actual, societal changes based on their academic work? The third step requires an *investigation into which ways of pursuing impact are acceptable and which are not, and to present the results as an ethical framework that reflects the scope and content of ethical challenges potentially encountered when pursuing impact.* To discuss what ways of pursuing impact (identified in step two) are acceptable or not, as well as identifying grey areas, will need to take place against a background of different theories. In the following we wish to draw attention to the variety of theoretical approaches that can be relevant for the bundle of normative theories that can be used to support the ethics for researchers who pursue impact of their research. Considerations about whether a piece of research is valid and worthy of being pursed calls e.g. accounts of multi-, interdisciplinary- and discipline transcending knowledge [10], and accounts for the relationship between expert and lay knowledge [27]. To respond to the question whether the conditions for the decision-making processes surrounding the efforts to produce impact is fair, we can draw upon theories addressing gender, equality and discrimination issues in general, and in health in particular, as well as the body of literature on stakeholder inclusion [28]. Just as external economic and political interests (e.g. pharmaceutical industry) are perceived as not always being legitimate drivers of research [29], merely promoting personal gains of researchers reflects questionable motivations. Moreover, we can find normative support for distinguish ethical acceptable from non-acceptable pursuit of impact in the research ethics literature in general (including codes and standards for researcher behaviour and the theoretical foundations of the RRI movement [30]), theories on legitimate, political decision-making [31], critical perspectives on unjustified stakeholder involvement [32], analysis of power [33] and ways to minimise unwarranted use of it, perspectives on trust in society and research [34, 35], and fairness in policy-making in health [36] and ethical theory of virtues [37]. Moreover, the discussion about a descriptive model of the researcher’s activities versus a more policy-engaging model has been central within anthropology since mid-90s [38]. This debate was inspired by a post-modern turn to a radical self-reflection of the role of the researcher and the dominance of “Western” Enlightenment models when dealing with the “other”. An approach to the specific ethical dimension of this transition can be discussed in relation to this debate, as well as in relation to literature on power (e.g. [33]).

#### Step 4

Suggesting that yet another research ethics framework is needed might imply a desire to impose yet greater control over research, and thus limit researcher autonomy. To mitigate this risk, rationales for a new ethical approach to impact, as well as strategies for implementation and evaluation, cannot be too demanding. Developing an ethical approach, must itself be ethical, in the fundamental sense that all involved and potentially affected by the framework are treated as moral equals; they are invited to voice their concerns, offer criticism, and shape the result. To foster legitimacy within the research community, this suggested development of an impact-pursuing ethical approach requires also transparency with regards to normative assumptions and methodology, as well as being explicitly exposed to constructive criticism. To invite input and feedback and foster legitimacy among the public in general, popular dissemination must go hand in hand with the academic research that is required. Also, a meta-perspective is required in order to scrutinise how researchers should be involved in coordinating and developing this ethical approach; they must, we maintain, adopt a reflexive stance, and ask when they are in a position to make normative conclusions or not [8, 16]. So, the fourth step on this path to an impact-pursuing ethics would be *to organise scientific and popular dissemination of 1–3) as a meta-exercise to enable critical scrutiny and allow for conferred legitimacy among stakeholders (including researchers, research funders, members of research ethics committees, politicians, and members of the society in general) for the process to establish this new ethics.*

## Conclusion

We have proposed a four-step approach towards developing a new ethics for researchers who are pursuing impact through their research. Whilst ‘research impact’ has been considered extensively, the novelty of our suggestion lies in the need to draw attention to how the transition between researchers acting merely to impact the scientific community and researchers acting to impact on the society ought to be configured, including the ethical challenges that are inherent to this transition. Our stepwise strategy to establish this new researcher ethics calls for both theoretical and empirical research and cannot be carried out within one disciplinary approach, say philosophy, alone, but will require efforts from a multi-disciplinary team of researchers and advisors from the humanities and social sciences. In so doing, we will be better positioned to clarify what ought to happen at this critical intersection between research, ethics, and politics. Such efforts should be able to offer new and useful assistance to researchers, as well as research funding agencies, in choosing ethically acceptable, impact-pursuing projects. In order to provide the public with *justified reasons* (as opposed to potentially blind trust) to place trust in research, we need institutions to assist in defining the scope and substantive content of research related challenges and reduce the risk of unintentional harms. We also foresee that an ethics for researchers who pursuing impact can serve such a societal function. It can be used for critical assessment of researchers’ performance, and thus constitute a useful, albeit not sufficient, condition for fostering trust in researchers.

Further discussions of the proposed, assumingly fair conditions for developing this new researcher ethics among stakeholders are indeed welcome before putting the developing process into motion. Based on further deliberation and critical scrutiny of the conditions we suggest above, the proposed framework can be developed into a generic methodology for identifying limits to ethical acceptable pursuit of impact not only within bioethics, but across disciplines. Once brought to fruition, this methodology can be applied to establish ethical guidance for impact-focused research in other thematic domains as well, e.g. climate and welfare, and provides a basis for future proposals and research.

## Data Availability

Not applicable.

## Abbreviations

SDG: Sustainable development goals
RRI: Responsible research and innovation
